# 435. Outcomes for E484K Mutation Negative COVID-19 Patients Cohorted with E484K Mutation Positive COVID-19 Patients: A Retrospective Cohort Study

**DOI:** 10.1093/ofid/ofab466.634

**Published:** 2021-12-04

**Authors:** Fahad Buskandar, Amber L Linkneheld-Struk, Victoria R Williams, Adrienne Chan, Lorraine Maze Dit Mieusement, Natasha Salt, Jerome A Leis

**Affiliations:** 1 University of Toronto, Toronto, Ontario, Canada; 2 Sunnybrook Health Sciences Centre, Pickering, Ontario, Canada; 3 Sunnybrook Health Sciences Centre, Toronto, ON, Toronto, ON, Canada

## Abstract

**Background:**

The emergence of the E484K mutation of SARS-CoV-2 poses a risk of immune evasion but the risk of re-infection during acute infection is not well defined. Our aim was to assess the risk of re-infection among patients with existing acute E484K mutation negative COVID-19 infection who were exposed to an E484K mutation positive SARS-CoV-2 infected patient.

**Methods:**

We performed a retrospective cohort study of patients admitted with acute E484K negative COVID-19 infection and shared a hospital room with a patient who was E484K mutation positive during their period of communicability. The primary outcome was laboratory confirmed and/or clinical evidence of re-infection within the E484K negative population within 30 days of exposure and the secondary outcome was the 30-day risk of death or re-admission to hospital due to COVID-19.

**Results:**

We identified 41 patients who were E484K mutation negative who shared a hospital room with some of the identified 34 E484K positive patients. Six (14%) underwent repeat COVID-19 testing and remained E484K negative and none developed signs or symptoms of COVID-19 re-infection during the 30 days following exposure. The mortality rate was 7% (3/41) and re-admission rate was zero at 30 days from exposure.

**Conclusion:**

Despite the small sample size, we did not observe any evidence of re-infection among patients with COVID-19 who shared a hospital room with E484K positive patients during their acute infection. If necessary due to high hospital occupancy, patients with discordant E484K results can be safely cohorted in a shared room.

**Disclosures:**

**All Authors**: No reported disclosures

